# Autologous bone marrow-derived mononuclear cell therapy in Chinese patients with critical limb ischemia due to thromboangiitis obliterans: 10-year results

**DOI:** 10.1186/s13287-018-0784-6

**Published:** 2018-02-22

**Authors:** Jianming Guo, Lianrui Guo, Shijun Cui, Zhu Tong, Alan Dardik, Yongquan Gu

**Affiliations:** 1 Department of Vascular Surgery, Xuanwu Hospital Capital Medical University, Beijing, China; 2 Institute of Vascular Surgery, Capital Medical University, Beijing, China; 3 Section of Vascular Surgery, Vascular Biology and Therapeutics, Yale University School of Medicine, New Haven, CT USA

**Keywords:** Bone marrow, Mononuclear cell, Thromboangiitis obliterans, Critical limb ischemia

## Abstract

**Background:**

For patients with thromboangiitis obliterans (TAO), revascularization with bypass or angioplasty is frequently not feasible due to the poor outflow of the distal small vessels. We evaluated the long-term results of our experience treating patients with TAO with autologous bone marrow-derived mononuclear cells (ABMMNCs) to determine the safety and efficacy of ABMMNC therapy in patients with critical limb ischemia due to TAO.

**Methods:**

This was a retrospective chart review from a single university hospital vascular surgery center between January 2005 and July 2006. Patients were treated with smoking cessation and either aspirin (100 mg/day) alone or aspirin and ABMMNC injection according to patient preference. Groups were compared for demographics, clinical characteristics, and short-term and long-term results.

**Results:**

Of 59 patients with TAO who were treated, 19 patients elected aspirin alone and 40 patients elected aspirin and ABMMNC injection. No patients suffered perioperative complications and 49 (83%) patients remained smoke-free for 10 years. The 10-year amputation-free survival was 85.3% (29/34) in patients treated with ABMMNCs compared to 40% (6/15) in patients treated with aspirin alone (*p* = 0.0019). Ulcer area (*p* < 0.0001), toe-brachial index (TBI; *p* < 0.0001), transcutaneous oxygen pressure (TcPO_2_; *p* < 0.0001), and pain score (*p* < 0.0001) were also significantly improved with ABMMNC treatment, although there was no difference in mean ankle-brachial index (ABI; *p* = 0.806).

**Conclusions:**

In patients with critical limb ischemia due to TAO, ABMMNC treatment was safe and effective. ABMMNC treatment significantly improved amputation-free survival, ulcer healing, and pain, although there is no difference in ABI compared to treatment with aspirin alone.

## Background

Thromboangiitis obliterans (TAO), also known as Buerger’s disease, was initially described by Leo Buerger at Mount Sinai Hospital in 1908 [1]. Although tobacco consumption is a major etiological factor, the pathophysiological mechanisms underlying TAO are still unknown. Patients with TAO frequently develop distal ischemia; however, revascularization with bypass or angioplasty is frequently not feasible due to the poor outflow of the distal small vessels and, as such, smoking cessation remains the main treatment to date [2–4].

In 2002, Tateishi-Yuyama et al. reported that autologous bone marrow-derived mononuclear cells (ABMMNCs) were a safe and effective treatment for critical limb ischemia [5]. BMMNCs are a mixed population of single nuclear cells that include monocytes, lymphocytes, and hematopoietic progenitor cells. Until now, cell therapy remains a controversial treatment for patients with critical limb ischemia due to peripheral arterial disease, as the optimal groups of patients who might benefit from this therapy remain poorly defined [6]. We have previously shown that unselected patients with critical limb ischemia can respond favorably to injected cellular therapy, although numerous factors remain to be optimized, including optimal patient selection [7–10]. Patients with TAO are thought to be a reasonable group of patients for ABMMNC therapy, and short-term clinical benefits of ABMMNC treatment have been shown in patients with TAO [11–13]. However, the long-term results of this treatment have not been reported. Since most patients with TAO are young, long-term evaluation of ABMMNC treatment is important for understanding the value of this therapy. We evaluated the long-term results of our experience treating patients with TAO using ABMMNCs.

## Methods

### Study population

The demographic and clinical data of consecutive patients with TAO treated at the Vascular Surgery Department of Xuanwu Hospital, Capital Medical University, between January 2005 and July 2006 were retrospectively reviewed. Approval for the study was obtained from the Xuanwu Hospital review board and all patients provided written informed consent to participate in the study.

The diagnosis of TAO was based on the criteria proposed by Olin [14]: 1) onset before age 45; 2) current (or recent) history of tobacco use; 3) presence of distal extremity ischemia (infra-popliteal or infra-brachial) with symptoms of claudication, rest pain, ischemic ulcers, or gangrene, and documented by noninvasive vascular testing such as ultrasound; 4) exclusion of autoimmune or connective tissue diseases, hypercoagulable states, diabetes mellitus, and proximal sources of emboli; and 5) consistent arteriographic findings in the clinically involved and noninvolved limbs [14].

All patients with TAO who had critical limb ischemia with severe rest pain and who were not candidates for angioplasty or surgical revascularization were eligible for enrollment in the study. Exclusion criteria included: 1) presence of a hematological disease or hypercoagulable state; 2) presence of a rheumatological or immune system disease; 3) acute lower extremity ischemia; 4) family history of TAO; and 5) refusal to accept smoking cessation as a treatment. Patients were treated with smoking cessation and aspirin (orally, 100 mg/day). All patients were required to remain strictly free of any smoking; patients were excluded from the study during follow-up if they relapsed in their smoking as indicated by self-reporting. All patients were offered treatment with ABMMNC therapy; patients were followed regardless of whether they received ABMMNC therapy or not.

### Demographic and clinical data

A review of preoperative demographic data, surgery characteristics, and early and late clinical outcomes was performed; the Society for Vascular Surgery reporting standards were used for stratification of comorbid conditions [15]. Preoperative demographic data included age, sex, comorbid conditions, smoking status, and severity of limb ischemia, including ulcer information.

Laboratory testing was performed on all patients as part of their admission for ABMMNC therapy, or as an outpatient for those patients not receiving ABMMNC therapy. Testing included complete blood count, blood glucose, liver/renal function, erythrocyte sedimentation rate, C-reactive protein, rheumatoid factor, fibrinogen, serum homocysteine, hypercoagulability screen (protein C, protein S, and antithrombin III plasma levels), chest radiograph, electrocardiogram, and ophthalmologic examinations. During the hospital stay, local wound care and debridement were performed per routine hospital protocols; ischemic ulcers were documented by digital color photography.

### ABMMNC treatment

**Fig. 1:**
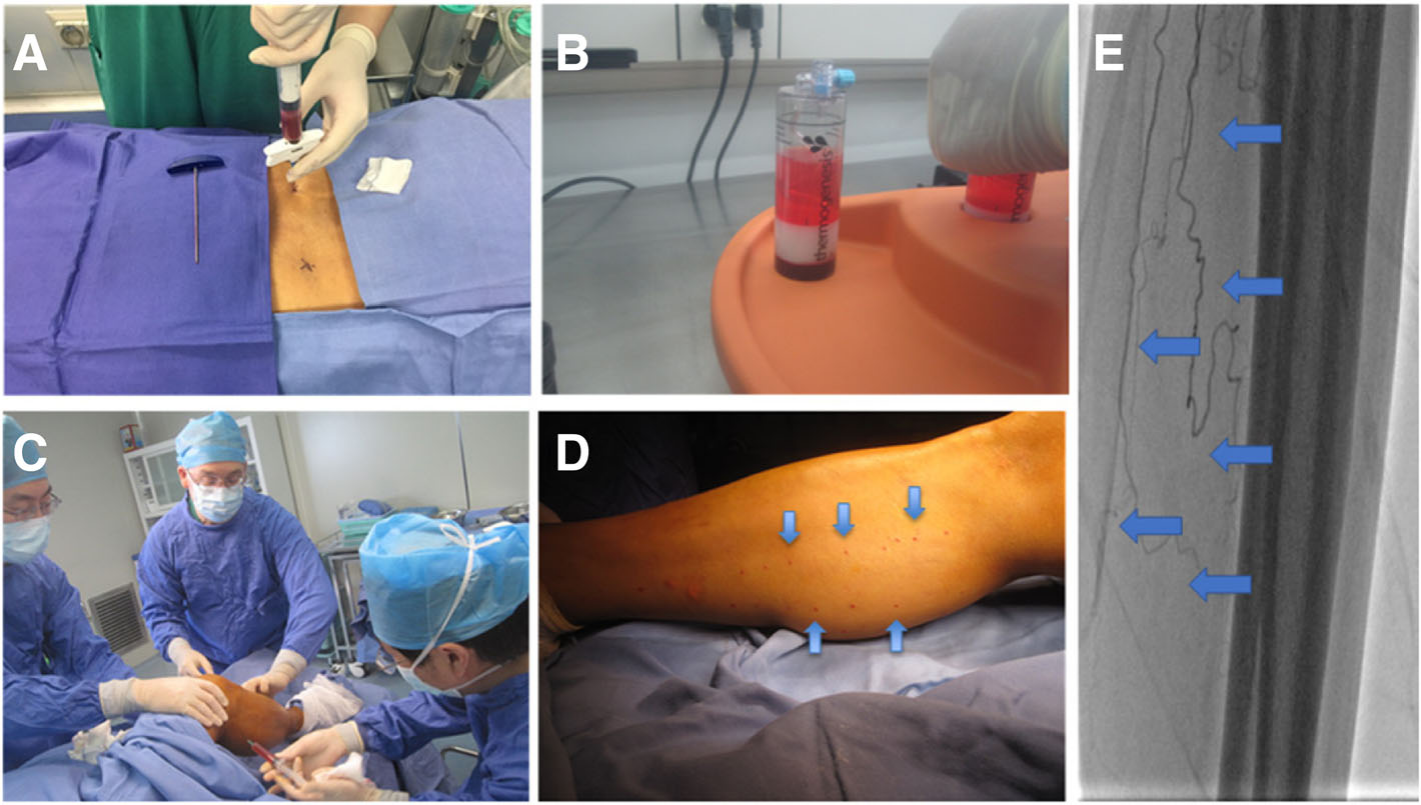
Representative images of ABMMNC treatment. **a** Bone marrow harvested from the posterior superior iliac spine. **b** Bone marrow separated into red cells and ABMMNCs. **c** ABMMNC injection. **d** Appearance of the leg after ABMMNC injections; blue arrows show the injection sites. **e** Representative angiogram, with blue arrows showing the injection sites along the tibial arteries

Antiplatelet agents were discontinued 7–10 days before ABMMNC treatment. Recombinant human granulocyte colony-stimulating factor (G-CSF; 300 μg/day) was subcutaneously injected for 3 days before the procedure [16]. In addition, heparin (12,500 units/day) was also subcutaneously injected to avoid thrombosis. Bone marrow aspiration sites were anesthetized with lidocaine, and then bone marrow was aspirated from the posterior superior iliac spines (Fig. 1a); routine harvest volume was approximately 200 ml. The collected bone marrow was immediately transferred to the laboratory. A continuous flow cell separator in a closed system (ZLDS Beijing Biotech Co., Beijing, China) was used to deplete red blood cells and concentrate the ABMMNCs for injection (Fig. 1b). Aliquots of the cell preparations were taken for testing for bacterial and fungal contamination; documentation of lack of contamination was required before injection. The same surgical team performed all procedures. Injection sites were selected based on the angiographic findings and were performed along the course of the arteries into the overlying muscle; injections were performed from 5 cm proximal to the obstructive lesion and continued distally to the upper edge of the malleolus. Each site was injected with 1 ml ABMMNCs (Fig. 1c–e).

### Endpoints and outcome evaluation

The primary clinical endpoint was amputation-free survival. Secondary outcomes included ulcer status, ankle-brachial index (ABI), toe-brachial index (TBI), transcutaneous oxygen pressure (TcPO_2_), and pain status. Amputation included both major amputation and minor amputation. Major amputation was considered to be either an above-the-knee or below-the-knee amputation, and minor amputation was considered to be a foot or toe amputation. TBI and ABI were measured with a dedicated machine (SmartdopXT, Hadeco, Japan). TcPO_2_ was measured with an oxymonitor (TCM400, Radiometer, Copenhagen, Denmark). Ulcer size was measured using the long × short axis and the area recorded (cm^2^). Improvement in ulcer size was noted when the ulcer area decreased to less than half of the baseline area. The pain score was measured using a visual analog scale (VAS); patients were instructed to place a mark on a line from 0 (no pain) to 10 (worst pain) that would best describe the level of their pain.

Major adverse events included death, Q-wave myocardial infarction, stroke, anemia, and unregulated angiogenesis such as clinically significant proliferative retinopathy or arterio-venous malformations. Malignancy was defined as biopsy-proven malignant transformation of any lesion in the leg.

### Follow-up

Patients were seen in the outpatient department at 1 month, 3 months, 6 months, and then annually for postoperative evaluation. Evaluation included examination and noninvasive testing as described above; retinopathy was monitored by funduscopic examination. Teratoma screening was performed by examining any radiology studies performed for clinical reasons for masses; amputation specimens were examined, especially near the margins. For patients without amputation, the absence of any abnormal mass was considered clinical evidence of lack of teratoma formation.

### Statistical Analysis

All data are presented as mean ± SD or frequencies. Differences between two groups were evaluated using the *t* test. Time-to-event analyses were performed using the Kaplan-Meier method. A *p* value < 0.05 was considered to be statistically significant. Statistical analysis software SPSS version 13.0 (Chicago, IL, USA) and Graphpad Prism version 5.0 (La Jolla, CA, USA) were used for statistical calculations.

## Results

### Demographics

Of 59 consecutive patients with TAO who were treated and offered ABMMNC treatment, 19 patients elected treatment with aspirin alone and 40 patients elected treatment with aspirin and ABMMNC treatment. Patients who did not want treatment with ABMMNC therapy typically had either a fear of stem cell therapy or an inability to pay for therapy.

The mean age of patients was 36.9 years; all patients were male. The baseline demographic and clinical characteristics, including hypertension, hyperlipidemia, diabetes mellitus, smoking history, and presence of an ischemic ulcer, were similar in both groups (Table 1). All patients had critical limb ischemia due to their TAO, with either rest pain, gangrene, or a nonhealing ulcer. The mean ABI, toe pressure, and TcPO_2_ were also similar in both groups (Table 1). Three patients had undergone prior femoral-tibial artery bypass grafting and three had undergone sympathetic ganglion block; these treatments were performed at least 1 year before ABMMNC treatment.

**Table 1:** Preoperative demographic characteristics

	Aspirin alone (*n* = 19)	Aspirin + ABMMNCs (*n* = 40)	*p* value
Male, *n* (%)	19 (100%)	40 (100%)	–
Age (years), mean ± SD	39.1 ± 4.2	36.2 ± 2.0	0.490
Hypertension, *n* (%)	1 (5.3%)	3 (7.5%)	0.749
Hyperlipidemia, *n* (%)	2 (10.5%)	3 (7.5%)	0.697
Diabetes, *n* (%)	0	2 (5%)	0.321
Smoking history, *n* (%)	19 (100%)	38 (95%)	0.321
Rest pain, *n* (%)	19 (100%)	40 (100%)	–
Gangrene, *n* (%)	5 (26.3%)	4 (10%)	0.103
Ulcer, *n* (%)	8 (42.1%)	24 (60%)	0.197
Ulcer area (cm^2^), mean ± SD	3.40 ± 0.96	3.70 ± 1.05	0.362
ABI, mean ± SD	0.45 ± 0.10	0.47 ± 0.11	0.607
Toe pressure (mmHg), mean ± SD	20.1 ± 2.1	18.5 ± 1.4	0.724
TBI, mean ± SD	0.16 ± 0.05	0.15 ± 0.04	0.584
TcPO_2_, mean ± SD	21.6 ± 5.2	22.3 ± 4.5	0.290
Previous bypass, *n*	0	3	0.221
Previous sympathectomy, *n*	1	2	0.964

*ABI* ankle-brachial index, *ABMMNC* autologous bone marrow-derived mononuclear cell, *TBI* toe-brachial index, *TcPO*_*2*_ transcutaneous oxygen pressure

### Perioperative period and short-term outcomes

All patients were treated with smoking cessation and aspirin. In the 40 patients who elected to be treated with ABMMNCs, the total blood volume aspirated from the posterior superior iliac spines was 212.5 ± 8.2 ml (range 200–230 ml) per patient, and the total volume of injected ABMMNCs was 43.4 ± 7.0 ml (range 40–45 ml) per patient. The total number of injected ABMMNCs was 3.5 ± 0.8 × 10^9^ cells (range 2.0–4.7 × 10^9^).

All injections were well tolerated. No patient suffered peri-procedural complications; there were neither hematomas nor any severe injection site pain afterwards. No patients received a transfusion after bone marrow harvest. The mean length of hospital stay for the treatment was 1.8 ± 0.7 days, and no patient required treatment in the intensive care unit.

### Long-term outcome

Of the 59 patients treated, 49 (83%) patients maintained strict smoking cessation for at least 10 years, 15/19 (79%) of the patients treated with aspirin alone and 34/40 (85%) of the patients treated with ABMMNCs (*p* = 0.563). Of the 10 patients that were unable to achieve strict smoking cessation and who were excluded from the study, nine patients relapsed into smoking during the first month and one patient relapsed at month 4.

In the 49 patients that were followed for 10 years, the mean follow-up time was 129.5 months (range 120–139 months); the mean follow-up time was 130.1 months in patients treated with aspirin alone and 128.3 months in patients treated with ABMMNCs (*p* = 0.613). No deaths, severe cardiovascular or cerebrovascular events, or malignancy occurred in any patient. All patients remained free of retinopathy and teratomas.

**Fig. 2:**
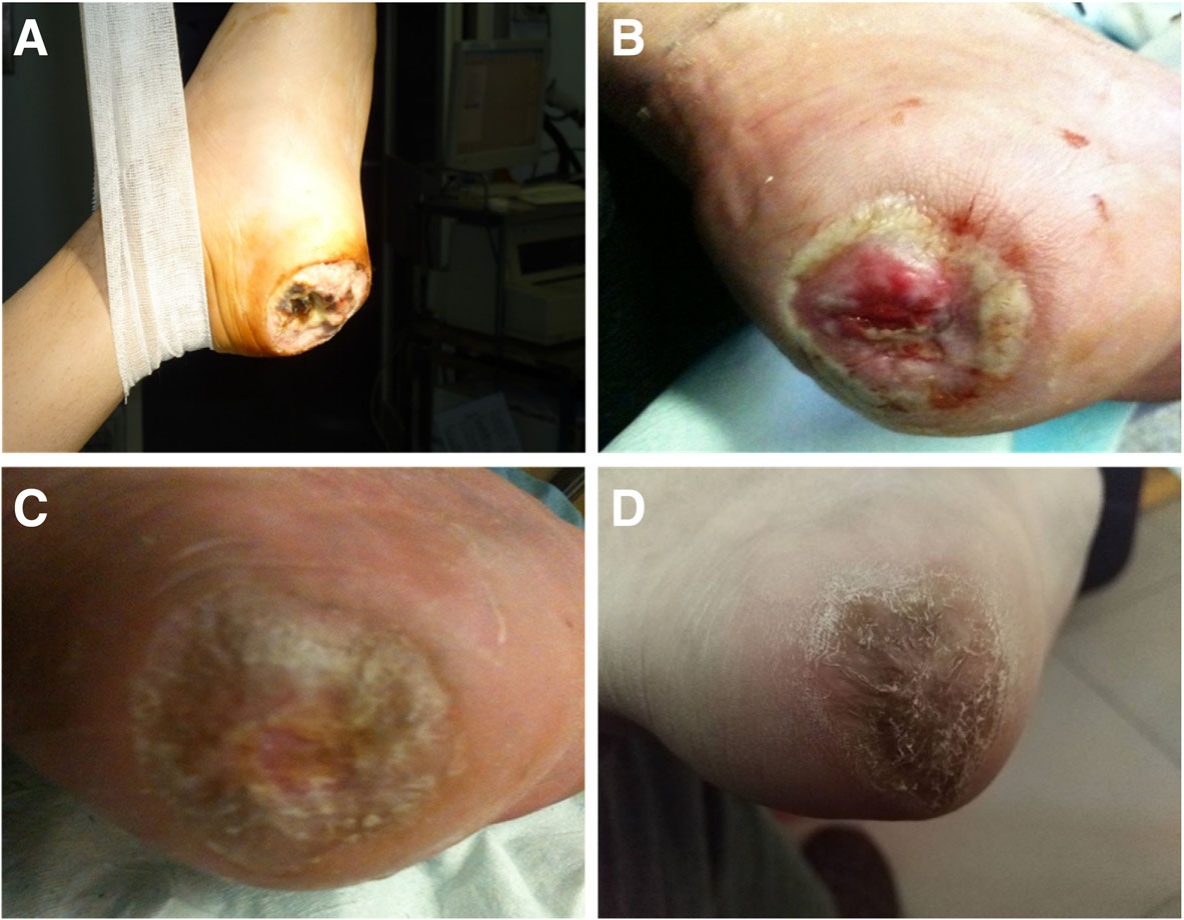
Representative images of an ulcer healing after ABMMNC treatment. **a** Prior to treatment; **b** at 3 months; **c** at 6 months; **d** at 120 months

**Fig. 3:**
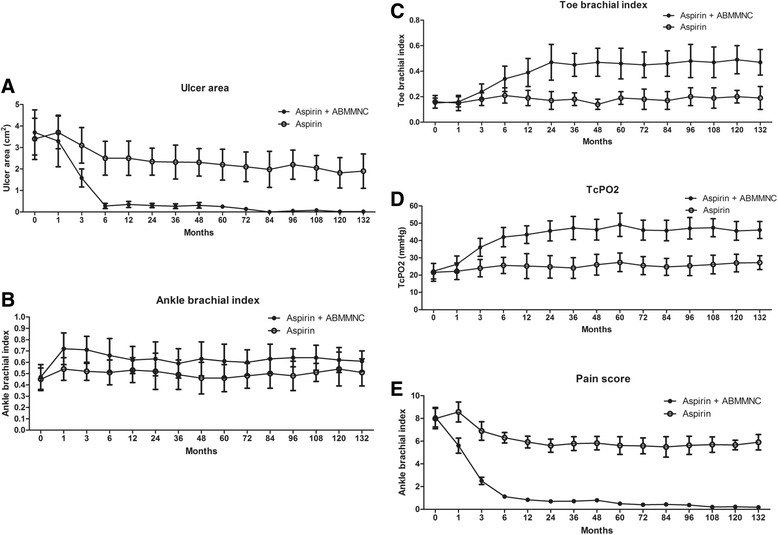
Time-dependent changes in **a** ulcer area (*p* < 0.0001), **b** ABI (*p* = 0.806), **c** TBI (*p* < 0.0001), **d** transcutaneous oxygen pressure (TcPO_2_; *p* < 0.0001), and **e** pain score (*p* < 0.0001). ABMMNC autologous bone marrow-derived mononuclear cells

Patients treated with smoking cessation and aspirin alone, without ABMMNC treatment, had a small improvement in ulcer area, and minimal improvements in toe pressure, TBI, TcPO_2_, and pain score over 10 years (Table 2). However, patients that were also treated with ABMMNCs had significantly greater improved ulcer healing at 1 year (0.35 vs. 2.5 cm^2^, *p* = 0.01) that was sustained at 10 years (0.0 vs. 1.8 cm^2^, *p* = 0.004; Figs. 2 and 3 and Table 2). Similarly, ABMMNC treatment resulted in sustained improvements in TBI (0.5 vs. 0.2, *p* < 0.0001), TcPO_2_ (46 vs. 27, *p* < 0.0001), and pain score (0.2 vs. 5.7, *p* < 0.0001; Fig. 3 and Table 2). Although there was a small increase in ABI over time in both groups of patients, there was no significant difference in ABI between the patients that received ABMMNCs and those that received aspirin alone (0.62 vs. 0.54, *p* = 0.215; Fig. 3 and Table 2).

**Table 2:** Patient data over time

		Baseline		1 year		5 years		10 years	
Ulcer area (cm^2^)	Aspirin alone	3.40 ± 0.96	P = 0.362	2.50 ± 0.80	P = 0.010	2.20 ± 0.72	P =0.007	1.82 ± 0.71	P = 0.004
	Aspirin + ABMMNC	3.70 ± 1.05		0.35 ± 0.14		0.25 ± 0.08		0.00 ± 0.01	
ABI	Aspirin alone	0.45 ± 0.10	P = 0.607	0.53 ± 0.11	P = 0.078	0.46 ± 0.12	P =0.090	0.54 ± 0.15	P = 0.215
	Aspirin + ABMMNC	0.47 ± 0.11		0.63 ± 0.12		0.61 ± 0.15		0.62 ± 0.11	
Toe pressure (mmHg)	Aspirin alone	20.1 ± 2.1	P = 0.724	23.8 ± 1.8	P<0.0001	23.4 ± 2.1	P<0.0001	25.2 ± 1.5	P<0.0001
	Aspirin + ABMMNC	18.5 ± 1.4		48.3 ± 5.1		56.1 ± 5.4		59.8 ± 6.6	
TBI	Aspirin alone	0.16 ± 0.05	P = 0.584	0.19 ± 0.06	P = 0.001	0.19 ± 0.05	P<0.0001	0.20 ± 0.05	P <0.0001
	Aspirin + ABMMNC	0.15 ± 0.04		0.39 ± 0.11		0.46 ± 0.12		0.49 ± 0.11	
TcPO_2_ (mmHg)	Aspirin alone	21.6 ± 5.2	P = 0.290	25.2 ± 7.2	P=0.0001	27.4 ± 5.4	P<0.0001	27.0 ± 5.1	P <0.0001
	Aspirin + ABMMNC	22.3 ± 4.5		43.4 ± 5.1		49.1 ± 6.7		45.6 ± 5.4	
Pain score	Aspirin alone	7.98 ± 0.90	P = 0.708	5.92 ± 0.52	P<0.0001	5.61 ± 0.78	P<0.0001	5.66 ± 0.42	P <0.0001
	Aspirin + ABMMNC	8.10 ± 0.86		0.84 ± 0.10		0.50 ± 0.06		0.22 ± 0.03	

ABI, ankle-brachial index; TBI, toe-brachial index; TcPO_2_, transcutaneous oxygen pressure

**Fig. 4:**
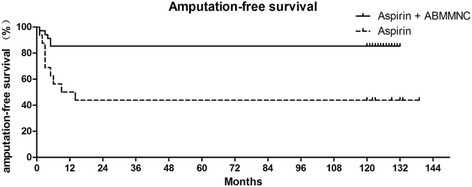
Amputation-free survival in patients treated with smoking cessation, aspirin, and with or without autologous bone marrow-derived mononuclear cell (ABMMNC) treatment; Kaplan-Meier analysis through 10-year follow up. Amputation includes both major and minor amputations

In patients treated with ABMMNCs there were no major amputations and only five minor amputations, whereas in patients treated with aspirin alone, two patients required below-knee amputations (χ^2^ = 5.749, *p* = 0.017), and seven patients required minor amputations (χ^2^ = 4.726, *p* = 0.030); patients treated with ABMMNCs had overall reduced numbers of amputations compared to treatment with aspirin alone (χ^2^ = 10.463, *p* = 0.001). Interestingly, most of the amputations occurred in the first 14 months (Fig. 4). The 10-year amputation-free survival was 85.3% (29/34) in patients treated with ABMMNCs but only 40% (6/15) in patients treated with aspirin alone (log-rank test, χ^2^ = 9.631, *p* = 0.0019; Fig. 4).

## Discussion

Several studies have demonstrated that ABMMNC treatment for atherosclerotic peripheral arterial disease is a safe and effective strategy that achieves therapeutic angiogenesis and prevents amputation [17, 18]. In patients with TAO, several studies have reported that stem cell therapy induces development of new collateral vessels and improves ischemic symptoms in short-term studies [19, 20]. Our data demonstrate that stem cell therapy in patients with TAO has a 10-year amputation-free survival rate of 85.3%, with all five amputations being minor. In addition, improvements in ulcer area, TBI, TcPO_2_, and pain score remained significantly improved from the baseline and were significantly better than in the conservatively treated group, even after 10 years. Furthermore, there were no cases showing development of retinopathy or cancer. These data show the long-term safety and efficacy of ABMMNC therapy in patients with TAO. However, especially given the young age of these patients, strict life-time surveillance is required after ABMMNC therapy.

Previously published studies have suggested rare mortality or morbidity in patients with TAO who were treated with ABMMNCs [18–20]. Consistent with these previous reports, our data similarly showed no deaths or severe adverse events. It is likely that the young age and few cardiovascular risk factors in patients with TAO may contribute to the safety of ABMMNC treatment. Miyamoto et al. [13] reported one death in a patient who was 30 years of age at 20 months after successful ABMMNC treatment; as no autopsy was performed, the cause of this single reported death after ABMMNC treatment remains unknown. Other than this single mortality, most studies have shown that cell therapy in patients with TAO is associated with few severe adverse effects. Therefore, in assessing stem cell therapy in patients with TAO, we believe that mortality is not a good indicator of treatment safety or efficacy.

Matoba et al. demonstrated that angiogenic cell therapy using bone marrow-derived mononuclear cells is associated with 3-year improvement in limb ischemia, leading to an extension of the amputation-free interval and improved perfusion compared to patients with atherosclerosis [21]. Our data are the first published 10-year results of ABMMNC treatment in patients with TAO and show the long-term safety and efficacy of this treatment. Interestingly, most outcomes, such as ulcer healing, toe pressure, TBI, and TcPO_2_, were improved in the first few months after treatment and then maintained during long-term follow-up (Table 2 and Fig. 3); this observation suggests that future studies can use shorter observation periods to determine the effects of treatment as well as adverse events. We believe that long-term improvement of symptoms and perfusion parameters with ABMMNC treatment may be due to three factors. First, patients with TAO have fewer cardiovascular risk factors; unlike patients with atherosclerotic limb ischemia, patients with TAO rarely showed involvement of their coronary, carotid, and visceral vessels, potentially reducing their risk for myocardial infarction, stroke, or mesenteric ischemia, respectively. Secondly, smoking and tobacco consumption are major factors associated with the development of TAO; since tobacco may trigger an immune response or unmask a clotting defect, tobacco can incite an inflammatory reaction in the vessel wall [22]. All patients that participated in this study signed a stop-smoking agreement. Interestingly, the modest clinical improvements in patients that stopped smoking but were not treated with ABMMNCs shows that smoking cessation alone may not improve ischemia, but it is likely that smoking cessation is a critical factor enabling proper stem cell function. Thirdly, lower extremity exercise training contributes to improved maximum walking distance, peak oxygen uptake, and quality of life in patients with intermittent claudication [23]. Since most patients with TAO rarely had severe comorbidities and generally had better physical condition than typical patients with atherosclerosis, it is likely that the patients who were treated with ABMMNCs were able to tolerate exercise training, giving additional benefit.

Of all the parameters that we evaluated, only the ABI showed no significant change after ABMMNC treatment, although the mean ABI increased slightly compared to baseline (Table 2 and Fig. 3). These results are similar to those reported in other studies; for example, in the Therapeutic Angiogenesis by Cell Transplantation (TACT) study [21], ABMMNC treatment showed no improvement in ABI after 3 years. It is likely that the lack of ABI improvement during long-term follow-up is related to the distal small arterial thrombosis that characterizes TAO [24]. Therefore, to evaluate long-term therapeutic results in patients with TAO, toe pressure, TBI, or TcPO_2_ may be more suitable outcome measures rather than ABI alone.

The mechanisms underlying stem cell therapy are still largely unknown. Yamamoto et al. reported that endothelial progenitor cells isolated from patients with TAO displayed high expression of endothelial lineage molecules compared with their counterparts obtained from patients with atherosclerosis [25]. This suggests that ABMMNC therapy can deliver bone marrow-derived endothelial progenitor cells that can incorporate into the existing vasculature, increasing capillary density and angiogenesis via paracrine effects [26]. It is also possible that delivery of ABMMNCs into the lower extremity hypoxic environment enhances their survival and/or function, as hypoxia promotes expansion of stem and precursor cell populations [27, 28].

## Conclusions

This study shows that in patients with critical limb ischemia due to TAO, ABMMNCs improve amputation-free survival, TBI, TcPO_2_, ulcer size, and pain status, with durable results through a 10-year follow-up. These data suggest the safety and efficacy of stem cell therapy in this select patient population. However, life-time surveillance is likely to be required for this younger group of patients.

## Abbreviations

ABI: Ankle-brachial index
ABMMNC: Autologous bone marrow-derived mononuclear cell
G-CSF: Granulocyte colony-stimulating factor
TACT: Therapeutic Angiogenesis by Cell Transplantation
TAO: Thromboangiitis obliterans
TBI: Toe-brachial index
TcPO_2_: Transcutaneous oxygen pressure
VAS: Visual analog scale
